# High PGE_2_ Levels Inhibit the Migration of Dendritic Cells by Modulating the PKA–CREB Axis Instead of Epac1–Rap1

**DOI:** 10.1155/sci5/8715442

**Published:** 2026-05-14

**Authors:** Ge Diao, Jie Huang, Min Tian, Runbo Li, Jianxin Guo, Jian Han

**Affiliations:** ^1^ Department of Gynaecology and Obstetrics, Daping Hospital, Army Medical University (Third Military Medical University), Chongqing, China, tmmu.edu.cn

**Keywords:** CREB, dendritic cells, Epac1, inhibited migration, PKA, Prostaglandin E2, Rap1

## Abstract

The inhibited migration of dendritic cells (DCs) from lesion tissue to draining lymph nodes is a possible way for tumors to achieve immune evasion. In a previous study, we demonstrated that high concentrations of Prostaglandin E2 (PGE_2_), which were expressed in tumor microenvironments, inhibited DCs’ migration. However, the specific mechanism is not yet clear. The current study aims to use the murine bone marrow–derived DCs (BMDCs) to demonstrate the possible signaling pathway of PGE_2_. The mRNA and protein expressions of the Epac1–Rap1 axis and PKA–CREB axis were determined after administration of PGE_2_ on DCs. Then, the agonist and antagonist of these molecules were used to treat DCs. The migration capability of DCs was detected as well as the RhoA activation levels. The mRNA and protein of Epac1 were barely undetectable in DCs. The use of Epac1 agonists showed no impact on DCs’ migration capability and RhoA activation levels. The activation levels of Rap1 and protein expressions of Rap1a and Rap1b were not affected by PGE_2_ administration. The using of Rap1a and Rap1b antagonists demonstrated no impact on DCs’ migration capability and RhoA activation levels. Moreover, the PKA activation levels and phosphorylated CREB1 were increased by the administration of PGE_2_ on DCs. The application of a PKA inhibitor attenuated the effect of PGE_2_ in a 3D migration assay and in vivo experiment. These results suggest that the PKA–CREB axis, instead of Epac1–Rap1, probably associates with the intracellular signaling pathway induced by high PGE_2_ levels, which demonstrates an inhibitory effect on DCs’ migration. These findings can bring a different perspective on immunological surveillance of tumor progression.

## 1. Introduction

The main function of dendritic cells (DCs) is to sample the surrounding environment for foreign antigens and present these antigens on the cell surface to T cells. They play a critical role in coordinating immune responses through acting as messengers between the innate and the adaptive immune systems [1]. Once activated by injury or inflammatory stimuli, DCs migrate to the lymph node and activate T cells to differentiate, which induces a robust immune response [2]. Therefore, the migration of DCs to draining lymph nodes is extremely critical in specific antitumor immunity.

Prostaglandin E2 (PGE_2_) is a cyclooxygenase product of arachidonic acid released from membrane phospholipids and thus modulates various pathological and physiological processes, including tumorigenesis [3–5]. Moreover, PGE_2_ has been shown to be the key modulator of DCs functions, including migration capability [6]. Our previous study demonstrated that PGE_2_ served a dual role in regulating the migration capability of DCs [7]. High concentrations of PGE_2_ inhibited cell migration, whereas low concentrations exhibited the opposite effect. Meanwhile, various tumor microenvironments have been identified to produce high concentrations of PGE_2_, including cervical cancer. Our previous study identified that high concentrations of PGE_2_ expressed by cervical cancer inhibited DCs’ migration [8]. However, the specific molecular mechanism has not been elucidated.

The PGE_2_‐induced signaling pathways are various. The cellular effects of PGE_2_ are believed to be dependent on the four functionally distinct receptor subtypes, which are expressed on the cell surface [9]. Due to their different functions, PGE_2_ induced intracellular signaling pathways are much more complicated. Depending on types of cells and organizations, different molecules are involved [10]. As a guanine nucleotide‐exchange factor, Epac1 was believed to mediate the intracellular cAMP‐dependent signaling [11, 12]. Together with Rap1, the Epac1–Rap1 axis was active in a variety of cells, which regulated cell adhesion and motility [13, 14]. Moreover, the Epac1–Rap1 axis has been shown to improve DCs’ migration by the regulatory T cell‐derived adenosine [15]. On the other hand, PKA was also reported to be involved in the signaling pathway of PGE_2_ [16, 17]. The PKA–CERB axis mediated the effect of PGE_2_ on promoting the proliferation and migration of lung adenocarcinoma cell lines [18]. Therefore, the truth needs to be explored.

Using the murine bone marrow‐derived DCs (BMDCs), the current study aims to explore the intracellular signaling pathway induced by high PGE_2_ levels, which inhibit DCs’ migration. Such data can be helpful in the better understanding of the mechanism of tumor immune evasion. More importantly, the biological implications of these findings may bring a different perspective on immunological surveillance of tumor progression.

## 2. Materials and Methods

### 2.1. Cell Culture

DCs were isolated from mouse bone marrow as previously described with slight modifications [19, 20]. C57BL/6 mice (male; age, 6–8 weeks; weight, 20–25 g; *n* = 21) were provided by the Experimental Animal Center of Daping Hospital, Third Military Medical University. Briefly, bone marrow cells were flushed out from the femurs and tibias of mice and subsequently removed of red cells. The remaining cells were cultured with RPMI 1640 supplemented with 10% FBS, 100 U/mL penicillin, 100 μg/mL streptomycin, 20 ng/mL recombinant GM‐CSF (Peprotech), and IL‐4 (Peprotech). On Day 3, nonadherent granulocytes were gently removed, and fresh media were added. On Day 7, the immature DCs were stimulated with 1 μg/mL LPS for 24 h. On Day 8, matured DCs were collected to be ready for the following experiments.

### 2.2. siRNA of Rap1a and Rap1b

Gene silencing of Rap1a and Rap1b was performed in DCs using Rap1a siRNA (sc‐41853, Santa Cruz) and Rap1b siRNA (sc‐41855, Santa Cruz), using Lipofectamine 2000 transfection reagent according to the manufacturer’s instruction for 72 h of incubation.

### 2.3. 3D Migration Assay

DCs were embedded into a collagen matrix (BD Matrigel; BD Bioscience; final concentration 1.7 mg/mL; 0.45 × 10^6^ cells/mL) in migration chambers (Perfusion Chamber; Electron Microscopy Sciences). The remaining space of the chambers was filled with medium containing 200 ng/mL of CCL19. Migration of DCs was recorded by bright‐field time‐lapse video microscopy at 37°C using inverted microscopes (Observer Z1; Zeiss) fitted with 10 × objectives and Axiocam cameras (Zeiss), which was started 10 min later after injection. Cells were imaged at a frame rate of 2 min up to 61 frames. Computer‐assisted cell tracking was performed with custom‐written software (ImageJ). The average speed was calculated as the step length per minute for each cell. Thirty randomly selected cells were included in one experiment.

### 2.4. PCR Detection for Epac1

Each PCR reaction was performed at the volume of 20 μL, which contained 10 μL of 2 × Taq Master Mix (Vazyme, Nanjing, China) and 0.5 μL of 10 pmol forward primer and 10 pmol reverse primer, respectively. Primer sequences for Epac1 were demonstrated in Table 1. The primers for GAPDH were purchased from Sangon Biotech (No. B661304). The thermal cycling conditions were as follows: a prerun at 95°C for 3 min and 35 cycles with a 15 s denaturation step at 95°C followed by a 60°C annealing step for 30 s. The PCR products were visualized on 2% agarose gel.

**TABLE 1:** Primer sequences used for PCR amplification.

Primer name	Nucleotide sequence	
	Forward (5′–3′)	Reverse (5′–3′)
Epac1 Primer 1	AGC​AGA​GAT​GCC​CGA​CTT​AG	GGT​TAG​GGA​GCC​AAA​CAG​GT
Epac1 Primer 2	GAG​TGT​CCC​ACA​TCC​ACG​A	AGT​TCC​CGC​TGG​TTG​TCA​A
Rap1a	GAA​CCG​AGC​AAT​TTA​CAG​CA	CAA​CTA​CCC​GTT​CAT​CTT​CCA
Rap1b	GCG​ACT​TGG​AAG​ATG​AAA​GAG	AAT​TTG​CCG​CAC​TAG​GTC​A
CREB1 [21]	GCT​GGC​TAA​CAA​TGG​TAC​GGA​T	TGG​TTG​CTG​GGC​ACT​AGA​AT

### 2.5. Real‐Time Quantitative PCR Detection for Rap1a, Rap1b, and CREB1

Each PCR reaction was performed at the volume of 20 μL, which was performed using the LightCycler 96 system (Roche). All reactions of the same run were prepared from the same total mix, which contained 10 μL of 2 × QuantiNova SYBR Green PCR Master Mix (Qiagen, Hilden, Germany) and 0.5 μL of 10 pmol forward primer and 10 pmol reverse primer, respectively. Primer sequences for Rap1a and Rap1b were demonstrated in Table 1. GAPDH was used as a reference gene. Each sample was amplified in triplicate. The thermal cycling conditions were as follows: a prerun at 95°C for 2 min and 40 cycles with a 15 s denaturation step at 95°C followed by a 60°C annealing step for 30 s, according to the LightCycler manual. Then the PCR run went into the melting curve analysis in order to ensure the specificity of the amplification products.

### 2.6. Western Blotting

Western blotting was used to determine the protein expression levels. After treatment, cell lysates were prepared by collecting DCs in the lysis buffer. Cell lysates containing equal amounts of protein were subjected to a 10% SDS–PAGE. Proteins were transferred to nitrocellulose membranes and subsequently blocked in T‐TBS buffer containing 5% BSA. Blots were incubated with anti‐Epac1 (Abcam; ab109415), anti‐Epac1 (MCE; HY‐P80120), anti‐Rap1a (Abclonal; A0975), anti‐Rap1b (Sangon Biotech; D220264), anti‐CREB1 (Beyotime Biotechnology; AF6566), antiphospho CREB1 (Ser133) (Beyotime Biotechnology; AF5785), and anti‐α‐tubulin (Beyotime Biotechnology; AF2831) overnight at 4°C. The membrane was further incubated at room temperature for 1 h with the horseradish peroxidase‐conjugated secondary antibody.

### 2.7. Pull‐Down Assay for Rap1 Activity

The detection of activated Rap1 was performed according to the Active Rap1 Pull‐Down and Detection Kit (Thermo Scientific, Schwerte, Germany). Cells were collected and washed twice with ice‐cold PBS, then added with 0.7 mL lysis buffer to obtain the cell lysates. Subsequently, the lysates were incubated with a Rap1 binding domain‐GST fusion protein and subjected to a glutathione–agarose resin, resulting in precipitation of activated Rap1. The precipitates were subjected to regular Western blotting and analyzed by Rap1 antibody.

### 2.8. cAMP Measurement

Cellular cAMP was quantified using a cyclic AMP ELISA kit from Cayman Chemical (581001). The cell lysates were obtained according to the manufacturer’s protocol. Protein concentrations were determined using a BCA protein assay kit (Thermo Fisher Scientific).

### 2.9. PKA Kinase Activity

PKA kinase activity was assessed by the PKA kinase activity ELISA kit (ab139435, Abcam) according to the manufacturer’s instructions. For every single test, equal protein amounts were used for analysis, allowing for comparisons using optical density (OD) at 450 nm. Each sample was analyzed in triplicate.

### 2.10. RhoA Activity Analysis

The active level of RhoA was determined by RhoA GLISA assay (Cytoskeleton) following the manufacturer’s instructions. Briefly, cells were collected and washed twice with ice‐cold PBS, then added with ice‐cold lysis buffer provided with the kit to obtain the cell lysates. The protein concentration of each sample was measured by the Bradford assay. An equal amount of protein was used for analysis in a single experiment.

### 2.11. In Vivo Migration Assay

C57BL/6 mice (male; age, 8–10 weeks; weight, 23–28 g; *n* = 3/group) were injected on the left footpads with 1 × 10^6^ labeled DCs. DCs were labeled with Qtracker™ 705 Cell Labeling Kit (Thermo Fisher Scientific, Inc.) according to the manufacturer’s instructions. Animals injected with PBS were used as controls. The inguinal and popliteal lymph nodes were collected and dissected 48 h after injection of DCs, embedded in Tissue‐Tek OCT compound (Sakura Finetek USA, Inc.) and frozen in liquid nitrogen. Cryosections (8 μm) were cut using a cryostat (Leica Microsystems GmbH). The sections were dried and frozen at −20°C before use. The slides were fixed with acetone (15 min; 4°C) and counterstained with DAPI (5 min; room temperature). Following washing with PBS, the slides were mounted in 50% glycerol (in PBS) and examined using fluorescence microscopy (magnification, x400).

### 2.12. Statistical Analysis

The statistical analysis was performed using a *t*‐test or one‐way ANOVA followed by a Dunnett test. Differences were considered significant when *p* < 0.05.

### 2.13. Chemicals

PGE_2_ (10 μg/mL) and 8‐pCPT‐2′‐O‐Me‐cAMP (O‐Me‐cAMP) (2 μg/mL) were purchased from Sigma‐Aldrich. Forskolin (0.5 μg/mL) was purchased from Cayman Chemical. H‐89 (1 μg/mL) was purchased from Beyotime Biotechnology.

## 3. Results

### 3.1. Epac1 is Not Involved in the Intracellular Signaling of High PGE_2_ Levels

First, we observed the cellular expressions of cAMP on DCs exposed to high levels of PGE_2_. As demonstrated in Figure 1(a), PGE_2_ stimulates the expression of cAMP. Forskolin, which was able to increase the intracellular cAMP concentration, enhanced the inhibitory effect of PGE_2_ on DCs migration (Figure 1(b)). These results indicate that high levels of PGE_2_ induce intracellular signal transduction. According to previous reports, there were several potential proteins mediating the effect of PGE_2_. Therefore, we explored the protein–protein interaction (PPI) network among these molecules in the STRING database (Figure 1(c)). Both the Epac1–Rap1 pathway and the PKA–CREB pathway could be the potential mediators of PGE_2_ effects. We determined to validate the role of the Epac1–‐Rap1 pathway in the first place.

FIGURE 1cAMP mediates the signaling of high PGE_2_ levels. (a) cAMP expression levels determined by ELISA kits. DCs were treated with PGE2 for 12 and 24 h and with forskolin for 4 h. The statistical data are expressed as mean ± SE of three independent experiments (*n* = 3). ^∗^ *p* < 0.05 compared with ctl. (b) DCs’ migration determined by 3D‐migration assays. DCs were treated with PGE_2_ for 24 h and forskolin for 4 h. The data are representative of three independent experiments (*n* = 3). Values are mean ± SE. ^∗^ *p* < 0.05 compared with ctl. (c) The STRING database reveals the interacting proteins in the signaling of PGE_2_.

**Figure:**
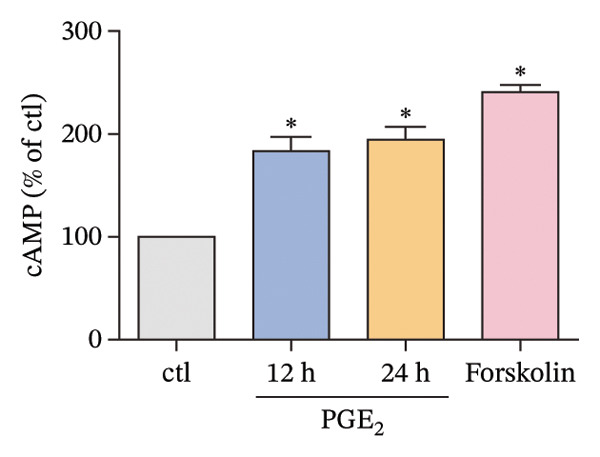
(a)

**Figure:**
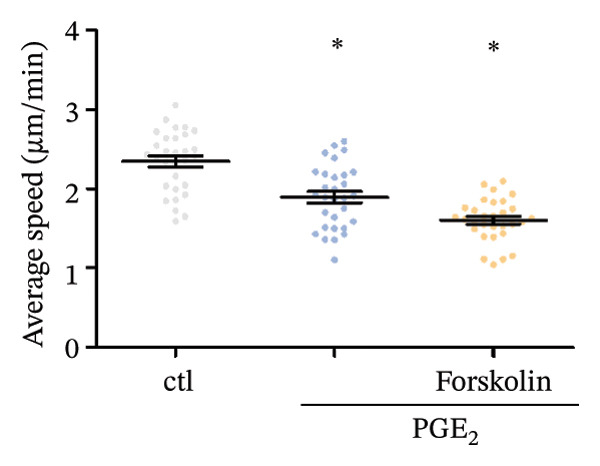
(b)

**Figure:**
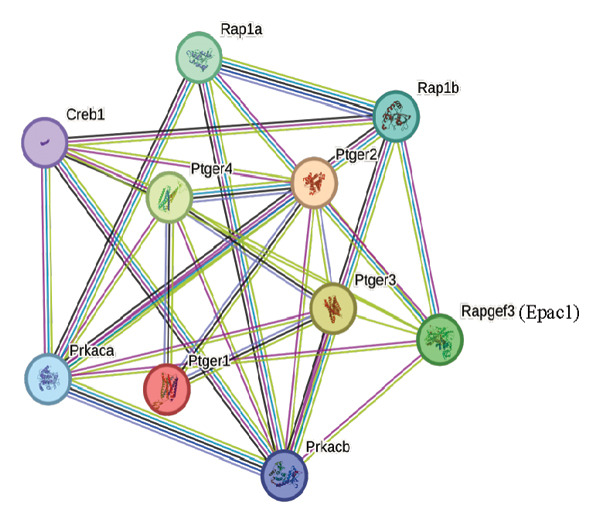
(c)

As depicted in Figure 2(a), the mRNA of Epac1 is barely undetectable in DCs using two different primer pairs, compared to its expression in mouse lung, which was reported in [22]. Then, the protein level of Epac1 was detected by Western blotting. Although up to 80 μg of total protein was loaded, the Epac1 protein was undetectable by using two different antibodies (Figure 2(b)). In the 3D migration assay, the stimulation on Epac1 by O‐Me‐cAMP (Epac agonist) failed to affect the migration ability of DCs (Figure 2(c)). Moreover, in order to explore the potential alteration of the cytoskeleton, we detected the activation of RhoA. As depicted in Figure 2(d), the activation level of RhoA is not changed by the treatment of O‐Me‐cAMP. These results indicate that Epac1 is not associated with the intracellular signaling of high PGE_2_ levels.

FIGURE 2Epac1 is not associated with the signaling of high PGE_2_ levels. (a) mRNA expression of Epac1 detected in DCs and mouse lungs by PCR. One representative gel image from three independent experiments is shown (*n* = 3). (b) Protein expression of Epac1 detected in DCs and mouse lungs by Western blotting. One representative blot from three independent experiments is shown (*n* = 3). (c) DCs’ migration determined by 3D‐migration assays. DCs were treated with PGE_2_ for 24 h and O‐Me‐cAMP for 4 h. The data are representative of three independent experiments (*n* = 3). Values are mean ± SE. ^∗^ *p* < 0.05 compared with ctl. (d) RhoA activation levels determined by the GLISA assay kit. DCs were treated with PGE_2_ for 24 h and O‐Me‐cAMP for 4 h. The statistical data are expressed as mean ± SE of three independent experiments (*n* = 3). ^∗^ *p* < 0.05 compared with ctl.

**Figure:**
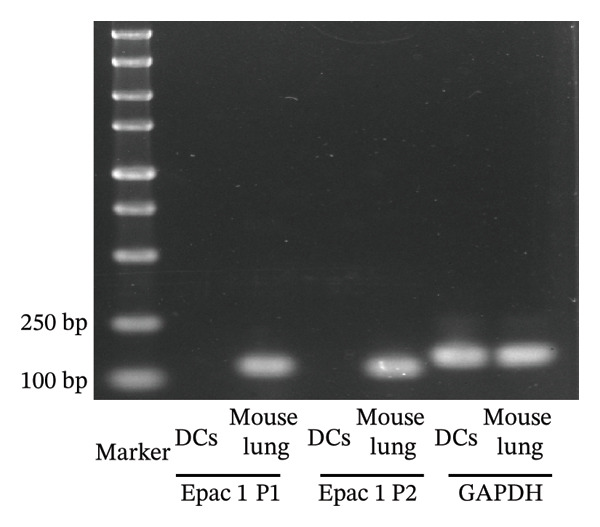
(a)

**Figure:**
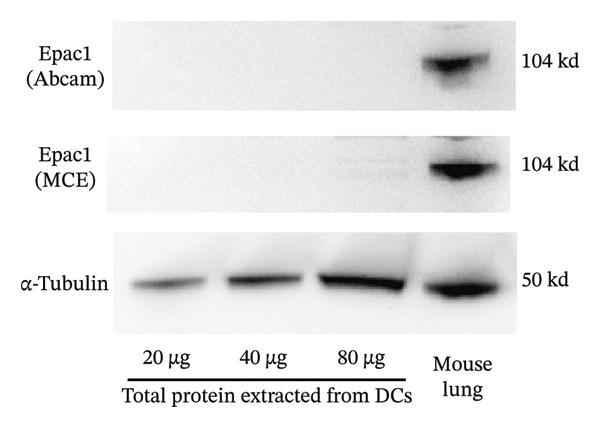
(b)

**Figure:**
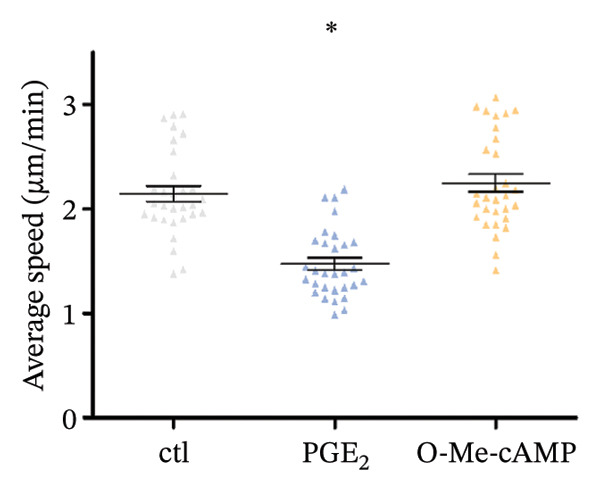
(c)

**Figure:**
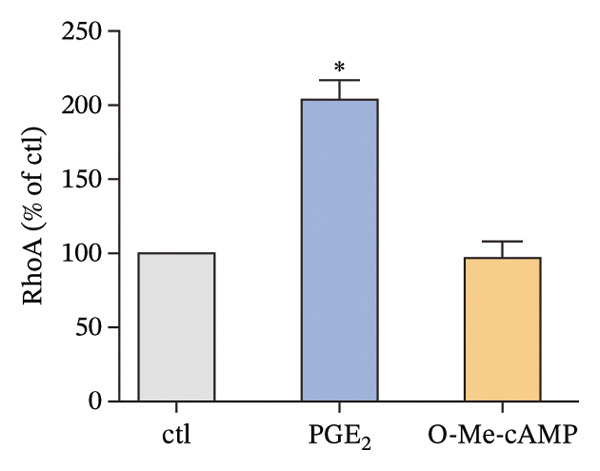
(d)

### 3.2. Activation of Rap1 is Not Associated With the Intracellular Signaling of High PGE_2_ Levels

The small GTP binding protein Rap1 is the major substrate of Epac1. Hence, we explored the potential role of Rap1 in the signaling pathway induced by high PGE_2_ levels. First, the pull‐down assays, followed by Western blotting, were performed to determine the activation level of Rap1 (Rap1‐GTP). The time course study showed that the activation levels of Rap1 were unaffected by the administration of high concentrations of PGE_2_ on DCs (Figure 3(a)). Since Rap1 exists as two separate isoforms, Rap1a and Rap1b, we tested them separately. The mRNA and protein expression of Rap1a and Rap1b were detected by RT‐qPCR and Western blotting, respectively. The mRNA expressions of Rap1a and Rap1b were both increased after PGE_2_ administration (Figure 3(b)). However, high levels of PGE_2_ showed no effect on the protein expressions of Rap1a and Rap1b (Figure 3(c)). The siRap1a and siRap1b were used as antagonists to interfere with the expression of Rap1a and Rap1b, respectively. The Western Blotting results confirmed that they functioned as expected (Figures 4(a), 4(b)). Subsequently, the 3D migration assay demonstrated that the application of siRap1a and siRap1b failed to attenuate the effect of PGE_2_ on DCs’ migration (Figure 4(c)). Moreover, as depicted in Figure 4(d), the activation level of RhoA is not changed by the treatment of either siRap1a or siRap1b in the presence of PGE_2_. These findings indicate that Rap1 is not associated with the intracellular signaling of high PGE_2_ levels.

FIGURE 3The effect of PGE_2_ on the expression of activated Rap1, Rap1a, and Rap1b. (a) Activated Rap1 determined by pull‐down assays. DCs were treated with PGE_2_ for 12, 24, and 48 h, respectively. One representative blot from three independent experiments is shown. The statistical data are expressed as mean ± SE (*n* = 3). (b) mRNA expression of Rap1a and Rap1b determined by real‐time quantitative PCR. DCs were treated with PGE_2_ for 12 h. The statistical data are expressed as mean ± SE of three independent experiments (*n* = 3). ^∗^ *p* < 0.05 compared with ctl. (c) Protein expression of Rap1a and Rap1b determined by Western blotting. DCs were treated with PGE_2_ for 24 h. One representative blot from three independent experiments is shown. The statistical data are expressed as mean ± SE (*n* = 3).

**Figure:**
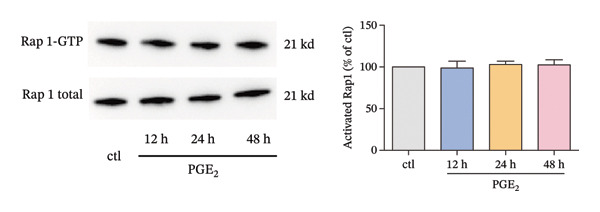
(a)

**Figure:**
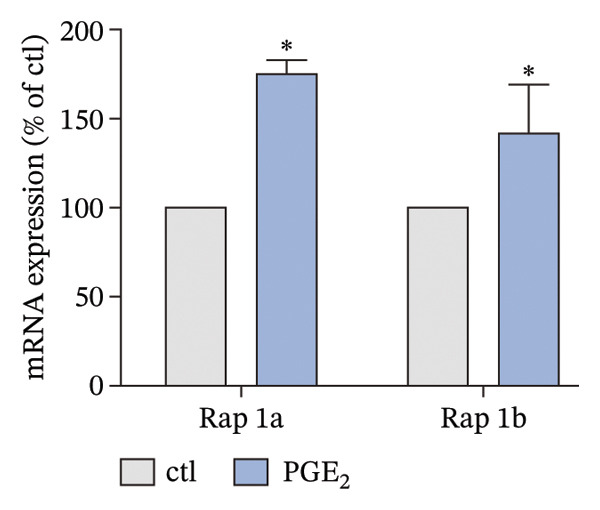
(b)

**Figure:**
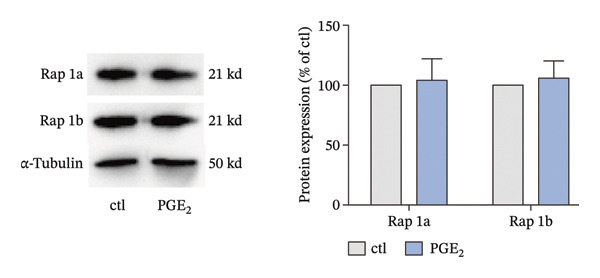
(c)

FIGURE 4Rap1a and Rap1b are not involved in the signaling of high PGE_2_ levels. (a) Protein expression of Rap1a determined by Western blotting. One representative blot from three independent experiments is shown. The statistical data are expressed as mean ± SE (*n* = 3). ^∗^ *p* < 0.05 compared with ctl. (b) Protein expression of Rap1b determined by Western blotting. One representative blot from three independent experiments is shown. The statistical data are expressed as mean ± SE (*n* = 3). ^∗^ *p* < 0.05 compared with ctl. (c) DCs’ migration determined by 3D‐migration assays. DCs were treated with PGE_2_ for 24 h after being transfected by siRNA. The data are representative of three independent experiments (*n* = 3). Values are mean ± SE. ^∗^ *p* < 0.05 compared with ctl. (d) RhoA activation levels determined by the GLISA assay kit. DCs were treated with PGE_2_ for 24 h after being transfected by siRNA. The statistical data are expressed as mean ± SE of three independent experiments (*n* = 3). ^∗^ *p* < 0.05 compared with ctl.

**Figure:**
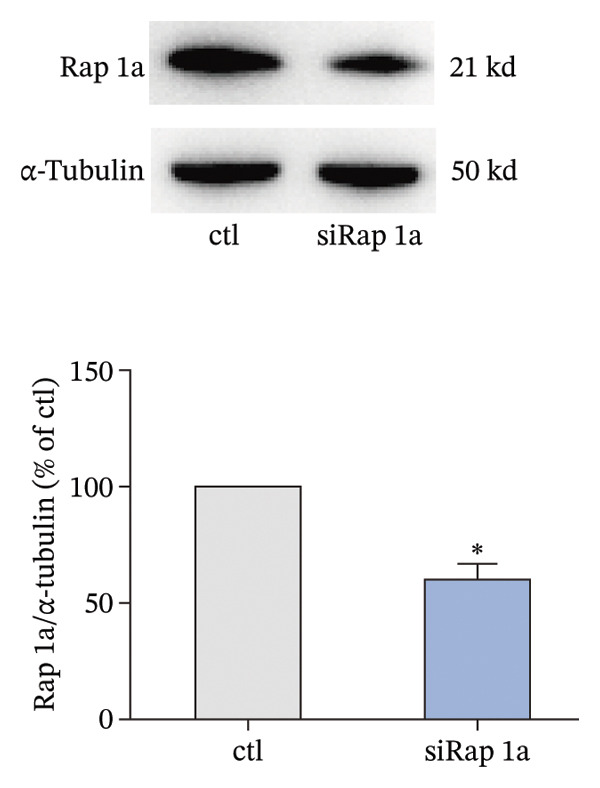
(a)

**Figure:**
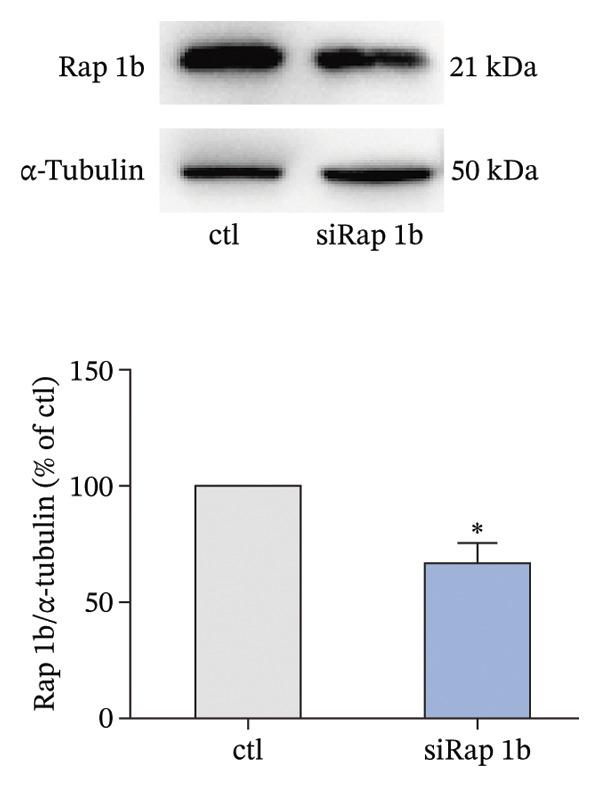
(b)

**Figure:**
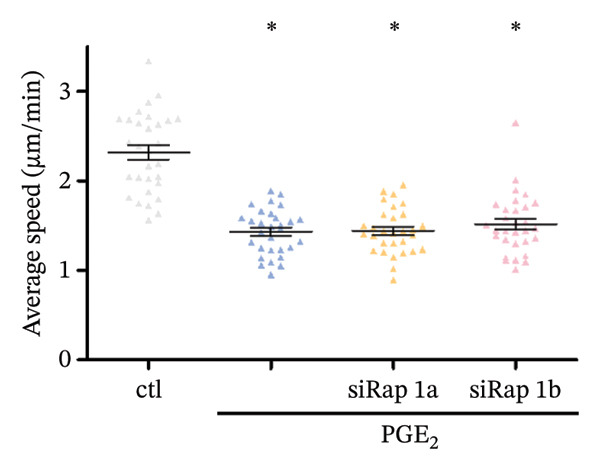
(c)

**Figure:**
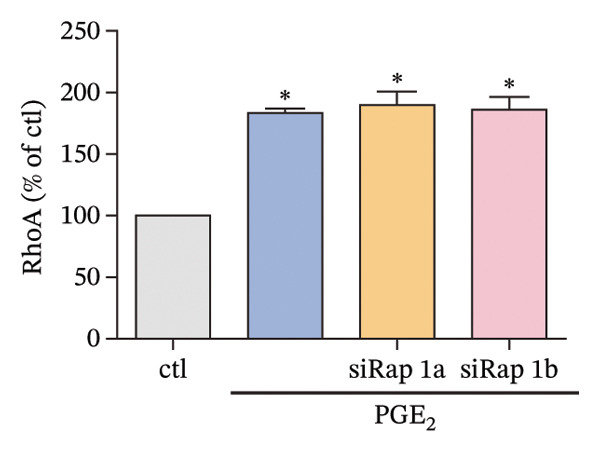
(d)

### 3.3. The Possible Involvement of PKA–CREB Axis in the Signaling of High PGE_2_ Levels

**FIGURE 6:**
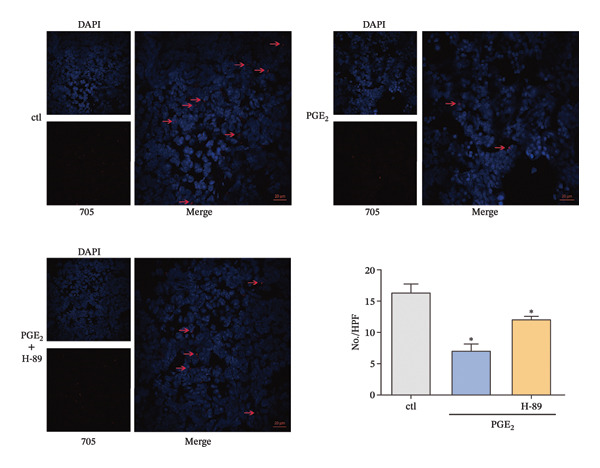
The in vivo experiments, which indicated that PKA is possibly associated with PGE_2_ signaling. Immunofluorescence microscopy of DCs in lymph nodes detected by confocal microscopy (original magnification, x400; scale bars, 20 μm). Red staining indicates Qtracker 705‐labeled DCs, while blue staining indicates nuclei. DCs were treated with PGE_2_ for 24 h and H‐89 for 4 h. One representative image from three independent experiments is shown. The statistical data are expressed as mean ± SE (*n* = 3). ^∗^ *p* < 0.05 compared with ctl.

Owing to the above results, we tried to explore other molecules that are potentially associated with the signaling pathway of high PGE_2_ levels. The activation level of PKA was detected. As demonstrated in Figure 5(a), high levels of PGE_2_ stimulate PKA activation on DCs. Subsequently, the expression of CREB1 was detected using a time course study. The mRNA expression of CREB1 was almost not influenced by PGE_2_ (Figure 5(b)). However, the administration of high levels of PGE_2_ increased the phosphorylation levels of CREB1 (Figure 5(c)). In the 3D migration assay, the inhibition on PKA by H‐89 (PKA inhibitor) attenuated the effect of PGE_2_ on the migration ability of DCs (Figure 5(d)). Furthermore, the role of PKA in the signaling pathway of PGE_2_ was validated with in vivo experiments using C57BL/6 mice. The numbers of labeled DCs, collected from the lymph nodes of mice, were compared among groups. These cells migrated from the footpads where they were injected. As demonstrated in Figure 6, H‐89 attenuates the effect of PGE_2_ on the numbers of labeled migrated DCs. These results indicate that the PKA–CREB axis may play important roles in the signaling pathway induced by high PGE_2_ levels.

FIGURE 5Expression of PKA/CREB1 is increased by high PGE_2_ levels. (a) PKA activation levels determined by PKA kinase activity ELISA kit. DCs were treated with PGE_2_ for 15 and 30 min, respectively. The statistical data are expressed as mean ± SE of three independent experiments (*n* = 3). ^∗^ *p* < 0.05 compared with ctl. (b) mRNA expression of CREB1 determined by real‐time quantitative PCR. DCs were treated with PGE_2_ for 15 and 30 min, respectively. The statistical data are expressed as mean ± SE of three independent experiments (*n* = 3). (c) Phosphorylated CREB1 determined by Western blotting. DCs were treated with PGE_2_ for 15 and 30 min, respectively. One representative blot from three independent experiments is shown. The statistical data are expressed as mean ± SE (*n* = 3). ^∗^ *p* < 0.05 compared with ctl. (d) DCs’ migration determined by 3D‐migration assays. DCs were treated with PGE_2_ for 24 h and H‐89 for 4 h. The data are representative of three independent experiments (*n* = 3). Values are mean ± SE. ^∗^ *p* < 0.05 compared with ctl.

**Figure:**
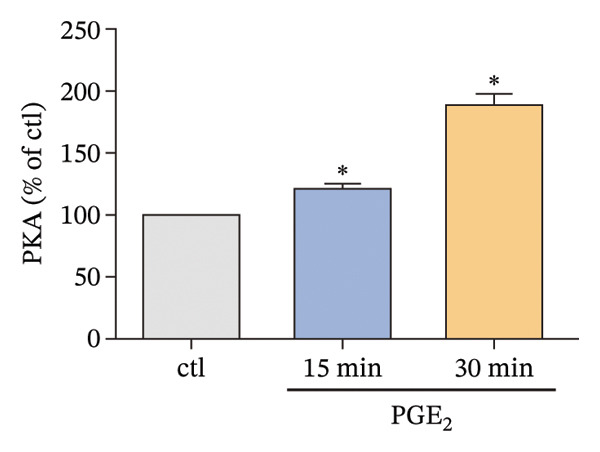
(a)

**Figure:**
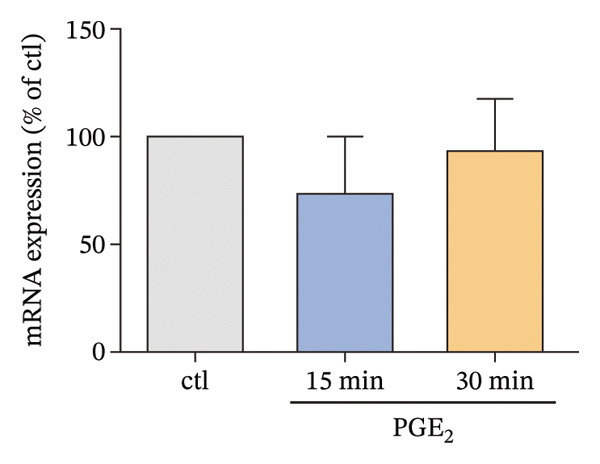
(b)

**Figure:**
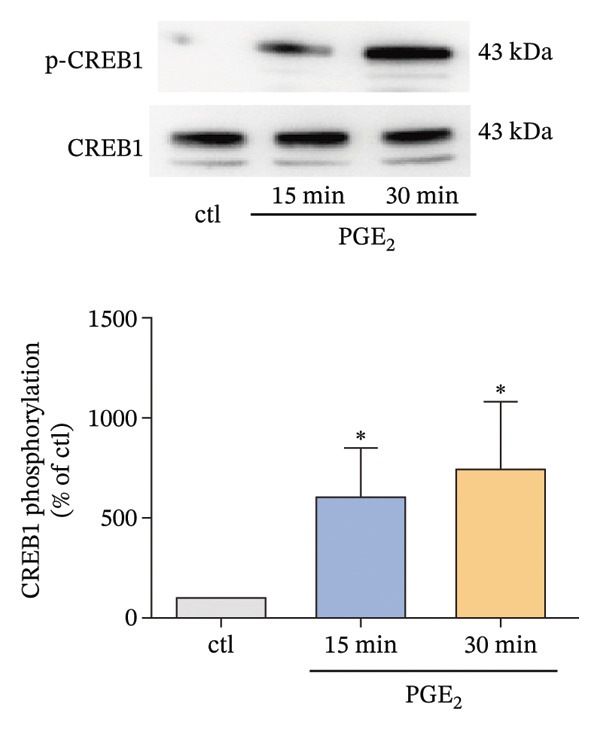
(c)

**Figure:**
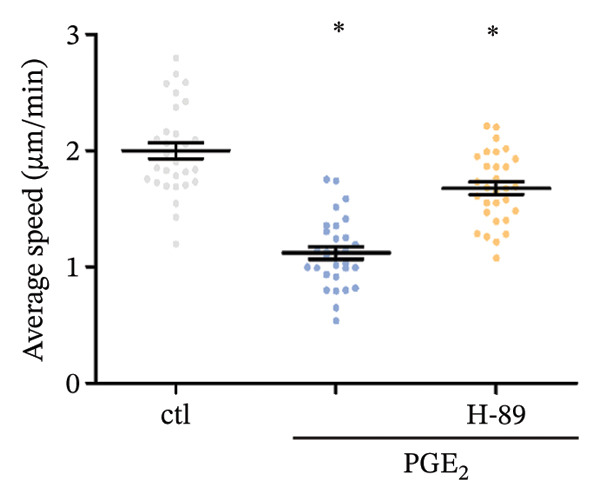
(d)

## 4. Discussion

PGE_2_ is the most widely studied member of the prostaglandin family, which exerts divergent modulatory action on the inflammatory responses. PGE_2_ signals through four morphologically distinct G‐protein‐coupled receptors (PTGERs) and elevates intracellular cAMP levels to trigger divergent signaling pathways, thereby mediating its various biological functions [23]. However, the PTGERs‐associated studies were not included in our research. We targeted the potential intracellular signaling pathways induced by PGE_2_. As a ubiquitous cellular second messenger, cAMP is known to regulate a wide range of cellular processes, including cell migration [24, 25]. Importantly, the effects of cAMP on migration can be variable, depending on the cell type. Kim et al. [26] reported that cAMP enhanced the migration of mouse embryonic stem cells (mESCs). In contrast, elevation of intracellular cAMP inhibited the migration of carcinoma cells [27]. Therefore, the action of cAMP on cell migration could be both stimulatory and inhibitory. In the current study, the intracellular cAMP demonstrated an inhibitory effect on the migration of DCs, which provided evidence for further exploration.

Actions of cAMP are mediated by multiple cAMP effectors, including PKA, Epac, PDZ‐GEF, and cyclic nucleotide‐gated channels. Notably, Epac exerted effector function upon GTP binding [28]. It has been reported that Epac1 suppressed the ability of PKA to induce the migration of DCs when both pathways were activated simultaneously [29]. Moreover, the PGE_2_ signaling has been shown to activate the Epac pathway independently without the activation of PKA [30]. These data seemed to support that Epac1, instead of PKA, mediated the signaling of high concentrations of PGE_2_. However, our results were not consistent with this. One possible explanation for this is that both PKA and Epac1 have dual roles. Although the inhibition on PKA inhibited migration of MDA‐MB‐435 breast carcinoma cells [31], it induced the migration of mouse embryonic fibroblasts [32]. Similarly, selective activation of Epac inhibited the migration of PC‐3 prostate carcinoma cells [33] and H295 adrenocortical carcinoma cells [34]. On the other hand, Epac promoted the migration of SK‐Mel‐2 melanoma cells [35]. Therefore, identifying their actual role in certain signaling pathways is very important to these studies related to cell migration.

An important finding in our experiments was that both mRNA and protein expressions of Epac1 in DCs were extremely low and almost undetectable (Figures 2(a), 2(b)). Such results are not in line with the findings described by Lorenowicz et al. [36]. They found that Epac1 proteins were expressed in all leukocytes except neutrophils. However, DCs were not directly included in their experiments. Therefore, our results can complement and modify this conclusion.

Meanwhile, another question arose. Rap1 was thought to control cell adhesion and the leading‐edge function of moving cells by interacting with and modulating adaptor proteins that help control the cytoskeleton [37]. However, our data showed that there was no direct evidence of a regulatory role for Rap1. There was a discrepancy between the protein and RNA expression level of Rap1a and Rap1b (Figures 3(b), 3(c)). This phenomenon has been clearly reported for specific genes in specific cell types. EEF1A1 has been reported to exhibit discordant mRNA and protein expression in ductal breast carcinoma [38]. There are many explanations for this phenomenon, and one important mechanism is post‐transcriptional regulation. It is very common for genes to exhibit inconsistent mRNA and protein expression as a result of post‐transcriptional regulation [39]. Therefore, post‐translational regulation may be the reason that Rap1 is not involved in the signaling pathway induced by high PGE_2_ levels.

Moreover, we demonstrated that the activation levels of RhoA were stimulated by the signaling of high PGE_2_ levels. RhoA is an important small GTPase, which is known to regulate the actin cytoskeleton in the formation of stress fibers [40, 41]. The active form of RhoA (RhoA‐GTP) regulated actin organization through its downstream target, the Rho‐associated coiled‐coil–containing protein kinase (ROCK) [42]. Furthermore, the active ROCK inhibited the generation of the F‐actin cytoskeleton to assist the cell migration [43]. Rap1 has been reported to modulate the activity of RhoA to affect cell migration capability [44]. However, our data showed that Rap1 failed to activate the expression of RhoA. Therefore, it can be considered that Rap1 does not play an important role in the signaling pathway of PGE_2_‐induced migration capability changes of DCs.

The relationship between PGE_2_ and the PKA/CREB pathway has been identified by many researchers. PGE_2_ was reported to promote the growth of T‐ALL cells by regulating the cAMP/PKA/CREB pathway [45]. PGE_2_ upregulated Aβ production through the PKA/CREB signaling pathway [46]. Moreover, PGE_2_ induced renal cell carcinoma (RCC) migration, which was associated with the G protein‐dependent CREB phosphorylation via PKA signaling [47]. Therefore, interrelating the PKA/CREB pathway with the inhibitory effect of high PGE_2_ levels on DCs’ migration is quite promising. However, in the current study, we only performed simple detection on PKA and CREB, which was inadequate due to time and labor limitations. They will be our main targets in future study.

There are several limitations that should be noted in this study. First, the in vivo experiment performed in this study may not fully reflect the authentic tumor microenvironment. This may prevent the effects of PGE_2_ from being fully represented. Therefore, validation in other models, such as orthotopic mouse models and patient‐derived tumor organoids, is needed to strengthen the conclusion. Second, due to the restriction on human tissue collection, we were unable to culture human DCs. Different cell origins may lead to variations in conclusions. This is another aspect that requires further validation.

## 5. Conclusion

Our data describe that the Eapc1‐Rap1 axis is not associated with the intracellular signaling pathway induced by high PGE_2_ levels, which inhibit DCs’ migration. Moreover, the PKA/CREB‐related pathways may be the potential molecules that mediate the effect of PGE_2_. Notably, these findings can be helpful in the better understanding of tumor immune evasion and contribute to new treatments for tumors.

## Author Contributions

Jian Han: conceptualization, funding acquisition, project administration, and writing–review and editing. Jianxin Guo: funding acquisition, conceptualization, and supervision. Ge Diao: writing–original draft, investigation, and methodology. Jie Huang: formal analysis and validation. Min Tian: data curation. Runbo Li: resources.

## Funding

This study was supported by the National Natural Science Foundation of China (grant no. 81272864) and Science‐Health Joint Medical Scientific Research Project of Chongqing (grant no. 2019ZY023464).

## Ethics Statement

Research related to animal use has complied with all the relevant national regulations and institutional policies for the care and use of animals and has been approved by the Laboratory Animal Welfare and Ethics Committee of the Third Military Medical University (Approval No. AMUWEC2019496).

## Consent

The authors have nothing to report.

## Conflicts of Interest

The authors declare no conflicts of interest.

## Data Availability

The data that support the findings of this study are openly available in Zenodo at https://doi.org/10.5281/zenodo.15804217.
